# Intrinsic neural timescales in autism spectrum disorder and schizophrenia. A replication and direct comparison study

**DOI:** 10.1038/s41537-023-00344-1

**Published:** 2023-03-30

**Authors:** Lavinia Carmen Uscătescu, Martin Kronbichler, Sarah Said-Yürekli, Lisa Kronbichler, Vince Calhoun, Silvia Corbera, Morris Bell, Kevin Pelphrey, Godfrey Pearlson, Michal Assaf

**Affiliations:** 1 Olin Neuropsychiatry Research Center, Institute of Living, Hartford, CT USA; 2 Centre for Cognitive Neuroscience & Department of Psychology, Paris-Lodron University of Salzburg, Salzburg, Austria; 3 Neuroscience Institute, Christian-Doppler Medical University Hospital, Paracelsus Medical University, Salzburg, Austria; 4 Department of Psychiatry, Psychotherapy & Psychosomatics, Christian-Doppler University Hospital, Paracelsus Medical University, Salzburg, Austria; 5 Tri-institutional Center for Translational Research in Neuroimaging and Data Science (TReNDS) Georgia State University, Georgia Institute of Technology, Emory University, Atlanta, GA USA; 6 Central Connecticut State University, Department of Psychological Science, New Britain, CT USA; 7 Yale University, School of Medicine, Department of Psychiatry, New Haven, CT USA; 8 University of Virginia, Department of Neurology, Charlottesville, VA USA

**Keywords:** Schizophrenia, Psychosis

## Abstract

Intrinsic neural timescales (INT) reflect the duration for which brain areas store information. A posterior–anterior hierarchy of increasingly longer INT has been revealed in both typically developed individuals (TD), as well as persons diagnosed with autism spectrum disorder (ASD) and schizophrenia (SZ), though INT are, overall, shorter in both patient groups. In the present study, we aimed to replicate previously reported group differences by comparing INT of TD to ASD and SZ. We partially replicated the previously reported result, showing reduced INT in the left lateral occipital gyrus and the right post-central gyrus in SZ compared to TD. We also directly compared the INT of the two patient groups and found that these same two areas show significantly reduced INT in SZ compared to ASD. Previously reported correlations between INT and symptom severity were not replicated in the current project. Our findings serve to circumscribe the brain areas that can potentially play a determinant role in observed sensory peculiarities in ASD and SZ.

## Introduction

Intrinsic neural timescales (INT) reflect the duration for which information is stored in specific brain areas^1,2^ and are instrumental to information processing in the brain^3^. In the human brain, INT length increases from posterior to anterior areas^4–6^. It has been suggested that caudal unimodal areas show shorter INT in order to enable the processing of fast, contextual changes^7,8^. On the other hand, the longest INT are found in anterior, higher cognitive brain areas, which perform the final integration and analysis of sensory inputs^4,9^.

A direct relationship between sensory peculiarities and INT was proposed by Zilio et al.^10^, who showed that people experiencing altered sensory states, such as unresponsive wakefulness syndrome or anaesthesia, also show prolonged INT. Information processing and integration are often atypical in clinical samples. Persons diagnosed with schizophrenia (SZ) or autism spectrum disorder (ASD) show atypical basic visual and auditory processing (e.g.^11–15^), as well as atypical object recognition^16^. Furthermore, autistic persons and those with SZ show atypical multisensory integration. For example, both groups show reduced proneness to perceive experimentally induced audio-visual illusions such as the McGurk effect (SZ^17^ ASD^18^). In addition, autistic persons show atypical habituation to joint auditory and tactile stimulation^19,20^, as well as opposite neural activation patterns during joint visual and auditory stimulation compared to TD^21^. In SZ, audio-visual integration was shown to negatively impact sound localization performance compared to TD^22^. Notably, atypical sensory integration in SZ has also been shown to be heritable^23^.

Indeed, recent studies suggest atypical INT in these clinical groups. In autistic persons, Watanabe, Rees, and Masuda^24^ found significantly shorter INT in the primary sensory regions (visual, sensorimotor, auditory) compared to TD. Similar findings in a sample of autistic adolescents within the same study suggested that there is a developmental component to INT patterns. Similarly, in SZ, our group^25^ found decreased INT in parietal and occipital areas compared to TD, which were also related to symptom severity. In addition, Wengler et al.^26^ showed that symptoms such as hallucinations and delusions are primarily related to alterations in somatosensory and auditory hierarchical INT gradients. Finally, Northoff et al.^27^ showed that INT of SZ are atypically prolonged during self-referential processes.

In the present study, we aimed to replicate previous findings regarding the INT patterns in autistic persons and those with SZ reported by Watanabe et al.^24^ and Uscătescu et al.^25^, respectively. Both studies rely on the same computational approach to define the INT of resting-state fMRI time series. Specifically, an autocorrelation function was calculated for each voxel at incremental time lags until its value became negative. The positive autocorrelation values were then summed up and multiplied by the repetition time (TR), thus resulting in the INT index.

Using resting-state data collected from both SZ and autistic persons at the same site and with an identical protocol, we applied a ROI analysis to focus specifically on the areas highlighted by these two studies to explore INT differences between each clinical group and controls. In light of the above-mentioned similarities in sensory processing between autistic individuals and SZ, and given previously documented overlap in underlying neuronal processes^28^, we directly compared the two patient groups and assessed the relationship between autism- and SZ-related characteristics and symptom severity and INT in these groups. Finally, we also performed exploratory whole-brain analyses to capture INT pattern characteristics of the three groups.

## Results

### Group differences in clinical and phenotypical assessment

Data collected from 55 TD, 30 ASD, and 39 SZ adults aged 18–35 (IQ > 80). Age (i.e., ASD < TD < SZ), estimated IQ (i.e., SZ < ASD < TD), and sex (i.e., more males than females) were significantly different between groups (Table 1). Therefore, these variables, as well as framewise displacement/FD (as described in the Methods section) were used as covariates in group-comparison analyses.

**Table 1:** Means and standard deviations (in parentheses) of demographics, phenotypic and clinical instrument scores for all three groups.

Demographic and assessment data							
	TD	ASD	SZ	ASD v. SZ v. TD	ASD > TD	ASD > SZ	TD > SZ
Females/males	29/26	5/25	8/31	**χ**^**2**^**(2) = 15.8, <0.000**			
				**F(2,121)**, ***p***	**t(df)**, ***p, p***_**FDR**_	**t(df)**, ***p, p***_**FDR**_	**t(df)**, ***p, p***_**FDR**_
Age (year)	24 (3.73)	22 (3.74)	26 (3.58)	**8.31, <0.000**	–1.98 (79.8), 0.051, 0.051	**−4.06 (76.9), <0.000, <0.000**	**−2.46 (88), 0.016, 0.024**
FD (mm)	0.08 (0.03)	0.09 (0.04)	0.11 (0.1)	**5.23, 0.007**	2.52 (66.6), 0.014, 0.021	**−0.772 (69.6), 0.443, 0.443**	**−2.71 (54.3), 0.009, 0.021**
est. IQ	112.26 (14.62)	109.1 (15.21)	99.41 (13.34)	**9.366, <0.000**	–1.33 (82.8), 0.186, 0.186	**3.26 (77), 0.002, 0.003**	**4.97 (90.5), <0.000, <0.000**
ADOS	1.87 (1.45)	10.1 (2.61)	8.41 (5.26)	**78.26, <0.000**	**17.5 (52.2), <0.000, <0.000**	1.67 (58.8), 1.72, 1.72	**−7.62 (44.2), <0.000, <0.000**
PANSS Positive		12.1 (2.86)	15.36 (4.86)			**−3.88 (66.96), <0.000**	
PANSS Negative		15.57 (4.7)	19.26 (6.2)			**−2.644 (72.75), 0.01**	
PANSS General		26.7 (5.62)	31.59 (6.98)			**−3.716 (73), <0.000**	

Group statistics are shown in the last four columns. Pairwise comparisons were performed using Welch’s two-sample *t*-test. Both uncorrected (i.e., *p*) and false discovery rate corrected (i.e., *p*_FDR_) *p* values are shown.

*FD* framewise displacement, *est. IQ* estimated Intelligence Quotient.

The group differences which survived FDR correction are written in bold.

### ROI replication results

We considered 13 ROIs for the current project, eight based on Watanabe et al.^24^ and abbreviated with the prefix “W_”, and five based on Uscătescu et al.^25^, and abbreviated using the prefix “U_” (Table 4). The INT were calculated as described in Watanabe et al.^24^, and based on the autocorrelation function of each voxel (please see the “Data analysis” section for more details).

First, we explored overall group differences by running an ANCOVA analysis with age, sex, IQ, and FD as covariates (summarized in Table 2). Of the ROIs based on Watanabe et al.^24^, only the right middle insula (W_rMidIns) showed a significant main effect (F (2, 131) = 3.31, *p* = 0.04, ηp2 = 0.05), but not after false discovery rate (FDR) correction (*p*_FDR_ = 0.1). Of the ROIs based on Uscătescu et al.^25^, four out of five showed a significant main effect that also survived FDR correction (Table 2).

**Table 2:** Group differences in INT per ROI, calculated with ANCOVA with age, sex, IQ, and FD as covariates.

Group differences in INT per ROI								
		INT mean (sd)						
		TD	ASD	SZ	F(2,131)	*p*	*p*_*FDR*_	ηp2
1	W_rPCG	2.88 (1.36)	2.8 (1.16)	2.37 (1.21)	2.74	0.07	0.11	0.04
2	W_lPCG	2.83 (1.27)	2.7 (1.08)	2.44 (1.2)	2.85	0.06	0.11	0.04
3	W_rMTG	1.3 (0.8)	1.46 (0.99)	1.21 (1.22)	0.38	0.69	0.69	0.01
4	W_lMTG	1.85 (0.95)	2.07 (1.06)	1.66 (1.18)	1.49	0.23	0.3	0.02
5	W_rIOG	2.16 (1.12)	2.19 (1.39)	1.67 (1.18)	2.28	0.11	0.16	0.03
6	W_rIPL	2.15 (0.93)	2.25 (1.17)	2 (1.18)	0.46	0.63	0.68	0.01
7	W_rMidIns	2.12 (0.93)	1.98 (1.11)	1.65 (1.16)	3.31	0.04	0.1	0.05
8	W_rCaud	0.79 (0.67)	0.88 (0.95)	0.76 (1.06)	0.78	0.46	0.54	0.01
9	**U_rOccFusG**	2.57 (1.28)	2.37 (1.22)	2.2 (1.22)	**5.65**	**0.004**	**0.03**	**0.08**
10	U_lSupOccG	2.18 (1.2)	2.17 (1.2)	1.86 (1.13)	2.8	0.07	0.11	0.04
**11**	**U_rSupOccG**	**1.64 (1.1)**	**1.4 (0.94)**	**1.28 (1.17)**	**4.48**	**0.01**	**0.03**	**0.06**
**12**	**U_lLatOccC**	**2.48 (1.15)**	**2.26 (1.1)**	**1.73 (1.19)**	**4.78**	**0.01**	**0.03**	**0.07**
**13**	**U_rPostCenG**	**2.7 (1.45)**	**2.44 (1.44)**	**1.92 (1.36)**	**7.44**	**<0.000**	**0**	**0.1**

The effect size was calculated using partial eta squared (ηp2). False discovery rate *p* values (${{{p}}}_{{{{\boldsymbol{FDR}}}}}$) are also shown. Results in bold font reflect post-FDR significant comparisons. Please see Table 4 for the explanation of the ROI acronyms.

Further group-wise comparisons via Welch’s one-tailed, two-sample *t-*tests verified whether the group differences previously reported by Watanabe et al.^24^ and Uscătescu et al.^25^, comparing each clinical group to TD could be replicated in our current sample. We also directly compared the two patient groups (these results are summarised in Table 3). None of the ROIs based on Watanabe et al.^24^ showed significant INT group differences between TD and ASD. The right inferior occipital gyrus (W_rIOG) and the right middle insula (W_rMidIns) showed significantly increased INT in ASD compared to SZ, but did not survive FDR correction (Table 3). Replicable group differences were found for two of the ROIs from Uscătescu et al.^25^, namely, both the left lateral occipital cortex (U_lLatOccC) and the right post-central gyrus (U_rPostCenG) displayed significantly reduced INT in SZ compared to TD (Table 3). These two ROIs also showed significantly increased INT in ASD compared to SZ, but only before FDR correction (Table 3).

**Table 3:** One-tailed, Welch’s two-sample *t*-tests of INT per ROI, with and without FDR correction, with Hedge’s g effect size.

Two-sample Group Comparisons in INT per ROI													
	ROI	TD v. ASD				TD v. SZ				ASD v. SZ			
		t(df)	*p*_unc._	*p*_fdr_	g	t(df)	*p*_unc._	*p*_fdr_	g	t(df)	*p*_unc._	*p*_fdr._	g
1	W_rPCG	0.32 (89.52)	0.38	0.45	0.06	1.97 (91.66)	0.03	0.08	0.39	1.62 (78)	0.05	0.16	0.36
2	W_lPCG	0.52 (89.68)	0.3	0.43	0.1	1.56 (88.99)	0.06	0.11	0.31	1.04 (77.76)	0.15	0.22	0.23
3	W_rMTG	–0.84 (69.72)	0.2	0.43	–0.18	0.42 (63.91)	0.34	0.37	0.09	1.01 (76.17)	0.16	0.22	0.22
4	W_lMTG	–1 (75.21)	0.16	0.43	–0.21	0.85 (73.97)	0.2	0.26	0.18	1.63 (77.77)	0.06	0.16	0.35
5	W_rIOG	–0.14 (69.69)	0.44	0.48	–0.03	2.05 (83.4)	0.02	0.07	0.42	1.8 (74.71)	0.04	0.16	0.4
6	W_rIPL	–0.43 (68.69)	0.34	0.44	–0.09	0.71 (72.83)	0.24	0.28	0.15	0.96 (77.86)	0.17	0.22	0.21
7	W_rMidIns	0.63 (71.48)	0.26	0.43	0.13	2.15 (73.75)	0.02	0.07	0.45	1.31 (78)	0.1	0.22	0.29
8	W_rCaud	–0.55 (63.34)	0.29	0.43	–0.12	0.13 (62.62)	045	0.45	0.03	0.54 (77.73)	0.3	0.3	0.12
9	U_rOccFusG	0.78 (84.54)	0.22	0.43	0.16	1.46 (88.94)	0.07	0.11	0.29	0.63 (77.81)	0.27	0.3	0.14
10	U_lSupOccG	0.03 (81.46)	0.49	0.49	0.01	1.35 (89.12)	0.09	0.13	0.27	1.2 (77.08)	0.12	0.22	0.27
11	U_rSupOccG	1.16 (89.4)	0.13	0.43	0.23	1.59 (82.74)	0.06	0.11	0.32	0.54 (75.94)	0.3	0.3	0.12
12	**U_lLatOccC**	0.89 (78.92)	0.19	0.43	0.19	**3.14 (84.39)**	**0.001**	**0.01**	**0.64**	1.99 (77.68)	0.03	0.16	0.44
13	**U_rPostCenG**	0.9 (81.93)	0.19	0.43	0.19	**2.76 (89.48)**	**0.004**	**0.03**	**0.55**	1.65 (77.09)	0.05	0.16	0.37

Results in bold font reflect post-FDR significant comparisons. Please see Table 4 for the explanation of the ROI acronyms.

**Fig. 1:**
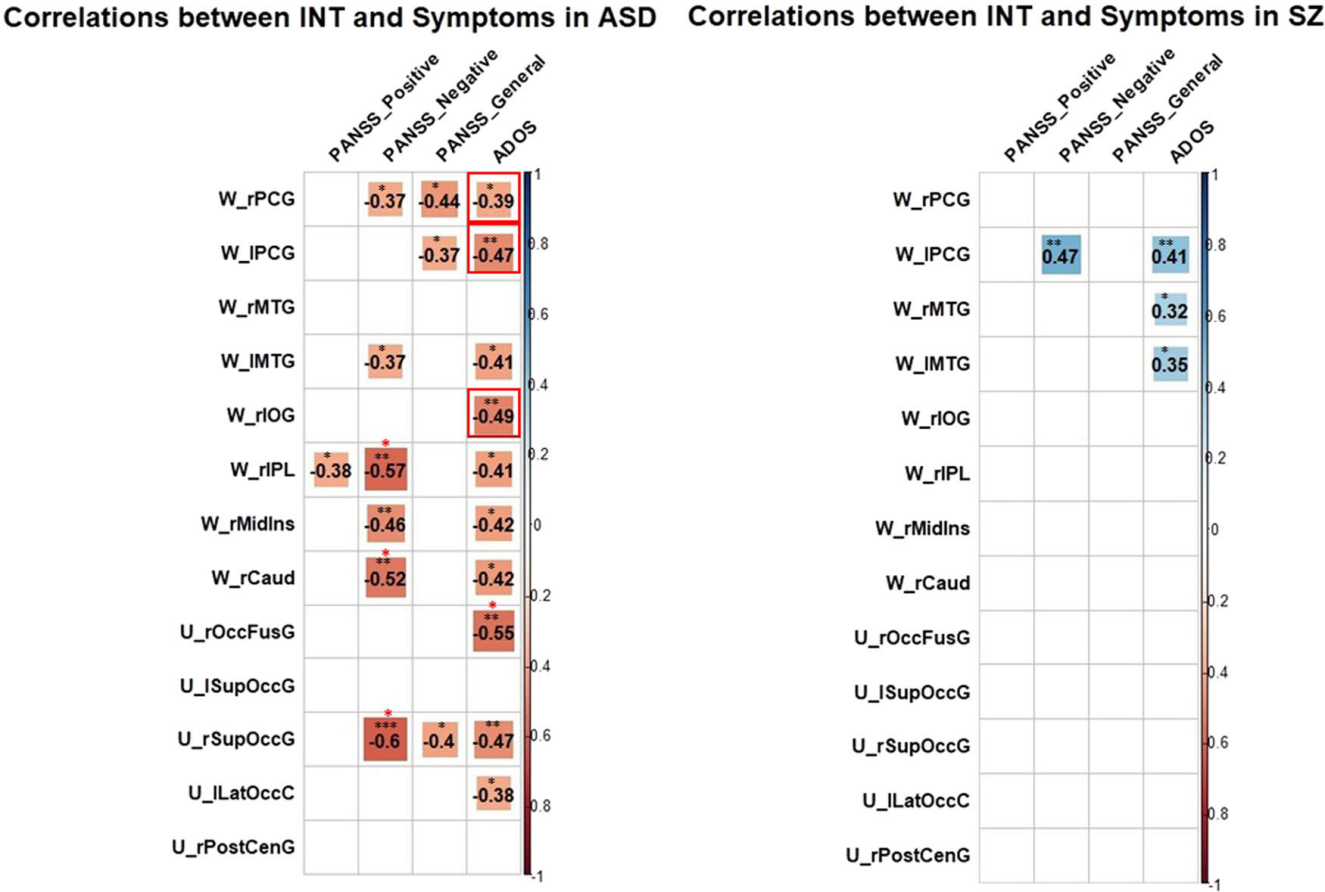
Spearman correlations between INT of ROIs and clinical measures in ASD (left) and SZ (right). Significant correlation values (*p ≤ .05, **p ≤ .01, ***p ≤ .001), both prior to (black asterisks) and after (red asterisks) FDR, are plotted, while non-significant correlations have been replaced by blank spaces. Replicated correlations are marked with red squares. Please see Table 4 for the explanation of the ROI acronyms.

Finally, we also assessed the relationship between clinical and phenotypic measures and INT in ASD and SZ, using Spearman correlations due to the INT variables not being normally distributed, according to the Shapiro-Wilk test (W > 0.7, *p* < 0.01 for ASD and W > 0.5, *p* < 0.01 for SZ). The significant correlations before (black asterisks) and after FDR correction (red asterisks) are shown in Fig. 1. None of the previously reported correlations between INT and symptom severity by Uscătescu et al.^25^ were replicated in the previous sample. In the ASD sample, we initially replicated the negative correlations reported by Watanabe et al.^24^ between the ADOS total score and the INT of the W_rPCG, W_lPCG and W_rIOG, but these did not survive FDR correction.

### Whole-brain exploratory results

**Fig. 2:**
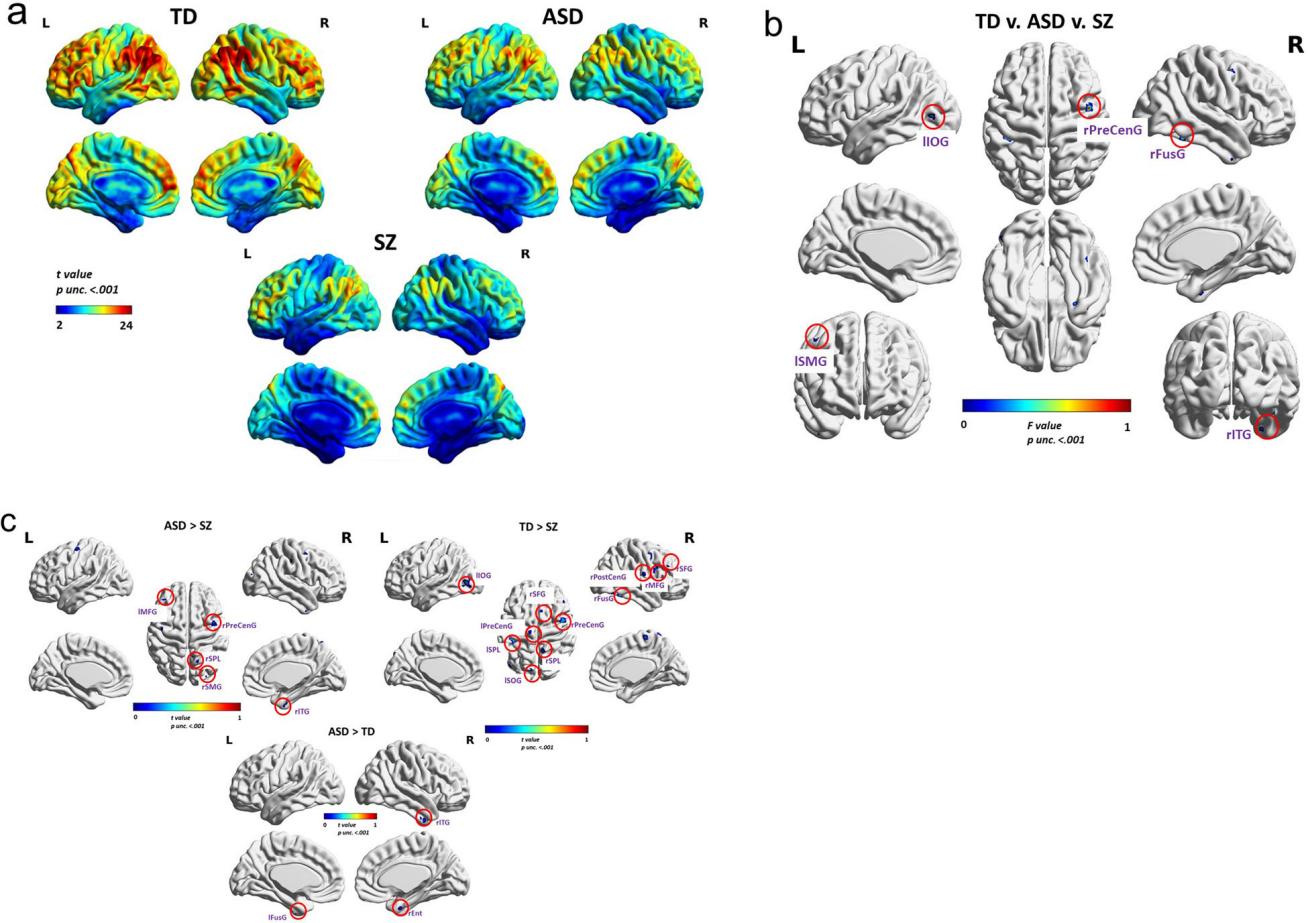
Whole-brain exploratory group comparisons. **a** Voxel-wise INT values within each group; TD typically developed, ASD autism spectrum disorder, SZ schizophrenia. *T* values on the scale bar range from 2 to 24. **b** Brain areas exhibiting INT differences across the three groups in a whole-brain ANOVA. *F* values on the scale bar range from 7.31 to 10.13. **c** Top left: Areas displaying higher INT in ASD compared to SZ. Top right: Areas displaying higher INT in TD compared to SZ. Bottom: Areas displaying higher INT in ASD compared to TD. *T* values on the scale bar range from 0 to 1. All results are for *p* unc. < 0.001. lLOG left Lateral Occipital Gyrus, lSMG left Supra-Marginal Gyrus, rPreCenG the right pre-central gyrus, rFusG the right fusiform gyrus, rITG the right inferior temporal gyrus, lFusG left fusiform gyrus, rEnt right entorhinal cortex, lMFG left medial frontal gyrus, lPreCenG left pre-central gyrus, rSPL right superior parietal lobe, rSMG right supra-marginal gyrus, rITG right inferior temporal gyrus, lIOG left inferior occipital gyrus, lSOG left superior occipital gyrus, lSPL left superior parietal lobe, rSFG right superior frontal gyrus, rMFG right middle frontal gyrus, rSFG right superior frontal gyrus.

In a first step, we performed mass-univariate analyses to compare brain-wise group differences in INT. Within-group INT are displayed in Fig. 2a. The areas that exhibited INT differences across the three groups (Fig. 2b) were the left lateral occipital gyrus (lLOG), the left supramarginal gyrus (lSMG), the right precentral gyrus (rPG), the right fusiform gyrus (rFusG) and the right inferior temporal gyrus (rITG). Pairwise comparisons showed no areas displaying higher INT in TD compared to ASD, but higher INT values were revealed in ASD compared to TD (Fig. 2c bottom) in the left Fusiform Gyrus (lFusG), rITG, and right Entorhinal Cortex (rEnt). Higher INT were found in TD compared to SZ (Fig. 2c top right) in the left Inferior Occipital Gyrus (lIOG), left Superior Occipital Gyrus (lSOG), left Superior Parietal Lobe (lSPL), left Pre-Central Gyrus (lPreCenG), right Superior Frontal Gyrus (rSFG), right Pre-Central Gyrus (rPreCenG), right Superior Parietal Lobe (rSPL), right Post-Central Gyrus (rPostCenG), rFusG, right Medial Temporal Gyrus (rMTG), and right Superior Frontal Gyrus (rSFG). No INT were found to be higher in SZ compared to TD. No areas displayed larger INT in SZ compared to ASD, but larger INT were found in ASD than in SZ (Fig. 2c top left) in the left Medial Frontal Gyrus (lMFG), rPreCenG, rSPL, right Supra-Marginal Gyrus (rSMG), and rITG.

## Discussion

In the current study, we sought to replicate previous findings regarding INT differences between TD and autistic individuals, and TD and SZ independently. We also verified to which extent previously reported relationships between INT and clinical and phenotypic characteristics could be replicated. In addition, we also directly compared autistic persons to SZ.

The ROI replication analyses of group differences, showed significant replication only for U_lLatOccC and the U_rPostCenG in the SZ group. However, other trends were preserved as discussed below.

Uscătescu et al.^25^ previously reported five ROIs that showed a trend of decreased INT in SZ compared to TD (right occipital fusiform gyrus/U_rOccFusG, left superior occipital gyrus/U_lSupOccG, right superior occipital gyrus/U_rSupOccG, left lateral occipital cortex/U_lLatOccC, right post-central gyrus/U_rPostCenG), two of which were replicated, in ref. ^25^, across three independent datasets, namely the U_lLatOccC and the U_rPostCenG. In the present study, we found a similar pattern of decreased INT in SZ compared to TD in all five ROIs, but this difference was significant only for U_lLatOccC and the U_rPostCenG. These results are in accord with previous reports showing increased temporal variability in SZ compared to TD in occipital and post-central gyri^29^, and in the right superior occipital gyrus^30^. While the methods employed by these authors differ from those used to quantify INT in our study, they converge onto a similar anatomical pattern of altered homeostasis with respect to information storage and processing in SZ. As it stands, there is significant interest in exploring these temporal dynamic processes in SZ through yet more diverse methodological approaches, such as dynamic Functional Network Connectivity (dFNC; e.g.^31^). A thorough interpretation of the clinical significance of these INT patterns in SZ can be found in ref. ^25^.

In addition, sensory areas have been shown to be hyper-connected to the thalamus in both autistic individuals^32^ and SZ^33^ compared to TD. In turn, hyperconnectivity between sensory areas and the thalamus have been linked to the severity of hallucinations and delusions in SZ^33^ and to general symptom severity in people with a diagnosis of ASD^32^. Taken together, this evidence suggests that the right post-central gyrus and superior occipital gyrus, in which INT group differences could be replicated in our study, reliably show atypical temporal patterns in SZ.

We observed similar trends to those reported by Watanabe et al.^24^ with respect to INT group differences between autistic persons and TD, namely that the former group had longer INT compared to TD in the right caudate/W_rCaud, and shorter ones in the bilateral postcentral gyri (i.e., W_rPCG and W_lPCG). However, none of the group differences survived FDR correction in our sample. We surmise that two aspects could have compromised the replicability of the results previously reported in their autistic samples by Watanabe et al.^24^. First, the reproducibility datasets used by these authors were very small: 25 (10 ASD) and 19 (9 ASD) respectively. Second, the lack of reproducibility of their results might have been due to the different preprocessing pipelines used by Watanabe et al.^24^ and Uscatescu et al.^25^ versus our current study.

In regards to symptoms correlations, no significant associations between SZ-related symptom severity, as measured by the PANSS, and INT were found in SZ group following FDR correction, thus we were unable replicate the correlations previously reported by Uscătescu et al.^25^. It is possible that this discrepancy can be attributed to SZ heterogeneity and/or to association of INT differences with different behavioral measure, not tested in this study.

With respect to the autistic group, the significant negative correlations between the ADOS total score, quantifying social-communication challenges, and the INT of the W_lPCG, W_rPCG, and the right inferior occipital gyrus/W_rIOG, previously reported by Watanabe et al.^24^, were initially replicated, but did not survive FDR correction. While Gratton et al.^34^ argue that when only small samples are available, brain-behavior relationships can be deemed reliable when out-of-sample replications are successful, which is the strategy that we have also employed in this project, these brain-psychopathology correlation results should be taken cautiously. Thus, we carefully speculate that these findings might indicate mechanistic effects, but emphasize the need to replicate them in a larger sample. According to Watanabe et al.^24^, who provide an in-depth discussion of these associations, these could further be attributed to the aetiology of autistic functioning and could be traced longitudinally.

We also directly compared the two clinical groups with respect to the INT of all 13 ROIs. In all cases, the INT of our autistic sample were longer than those of SZ, but not significantly, following FDR correction. We believe that these results are a relevant step towards localizing the brain areas that might be mechanistically involved in temporal irregularities and the resulting neural functioning. However, given the diagnostic heterogeneity characteristic of both autism and SZ, and the limited sample size of the current study, it is necessary to further ascertain the replicability of these findings before more decisive conclusions can be formulated.

Finally, as previously reported by Watanabe et al.^24^ and Uscătescu et al.^25^, a gradient of increasing INT from posterior to anterior areas was observed in TD, and preserved in both clinical groups. In accordance with previous reports, the overall INT were smaller in autistic individuals and SZ compared to TD (Fig. 2a). We further explored brain-wise group comparisons (Fig. 2b, c), but found only very small clusters which did not survive FDR correction. We believe that these initial exploratory results might nevertheless be helpful for future explorations, and perhaps with larger samples they might lead to more significant insight.

The present study has the distinct advantage that rsfMRI data from both clinical samples and controls were recorded in the same setting, thus eliminating potential confounds related to variation in data collection. However, some limitations remain. On the one hand, some sensory areas, like the postcentral gyrus, have been shown to display decreased sensory sensitivity with age^35^. Although we included age as covariate in our group analysis, age-balanced samples would have been optimal. A second limitation, which also determined our choice of not eliminating participants despite the age imbalance, is related to the relatively small sample sizes. The limited sample size also prevented us from assessing potential sex differences within and between the three groups. In addition, given the wide phenotypic variability of persons diagnosed with ASD or SZ, and more recent arguments in favor of subgrouping these heterogenous diagnostic entities into more homogenous sub-groups (e.g.^36–39^), acquiring larger samples is imperative for a thorough assessment of the relationship between INT and sensory processing in autism and SZ. Finally, an appropriate future direction would be to extend the current project to samples from which sensory processing measures have explicitly been collected; this was unfortunately not possible in the current project.

In conclusion, the present study is a step forward in assessing the replicability of INT characteristics in autism and SZ. Despite current sample size limitations, it appears that the left lateral occipital gyrus and the right post-central gyrus hold the highest promise concerning replicable INT group differences between SZ and TD. Substantially larger samples are still required to definitely assess the robustness of the relationship between INT and psychopathology.

## Methods

### Participants

Participants were recruited via the Olin Neuropsychiatry Research Center (ONRC) at the Institute of Living, Hartford Hospital, and the Department of Psychiatry, Yale School of Medicine and underwent resting-state fMRI scanning for the current study. Participants provided written informed consent and were paid for their participation. All procedures involved in this study were pre-approved by the Institutional Review Boards of Hartford Hospital and Yale University.

After discarding datasets displaying head motion > 10 mm, our dataset contained 58 TD, 39 ASD, and 41 SZ. Of these, some were subsequently excluded due to incomplete phenotypic assessment information, thus resulting in the following final samples: 55 TD, 30 ASD, and 39 SZ. As this dataset has been previously used in refs. ^40–42^), the exclusion criteria were the same, namely: intellectual disability (i.e., estimated IQ < 80), a neurological disorder (e.g., epilepsy), current drug use as indicated by pre-scanning interview and urine test, incompatibility with MRI safety measures (e.g., ferromagnetic implants), and a history of psychiatric and neurological diagnoses in TD.

### Clinical and phenotypical assessment

The severity of psychotic symptoms was assessed using the Positive and Negative Syndrome Scale (PANSS^43^) in both the ASD and SZ groups. The PANSS scores can be interpreted along three subscales: positive symptoms, reflecting the severity of hallucinations and delusions; negative symptoms, reflecting the severity of blunted affect and anhedonia, and a general subscale quantifying other psychopathologies such as poor attention and lack of insight. The ADOS, module 4^44^ was administered to all participants to confirm/rule out an ASD diagnosis. ADOS total score (social interaction and communication subscores) was used to measure autism-related symptom severity. The Intelligence Quotient (IQ) was estimated for the entire sample using the Vocabulary and Block Design subtests (according to ref. ^45^; also see ref. ^46^) of the Wechsler Scale of Adult Intelligence-III (WAIS-III^47,48^). The structured clinical interview for DSM-IV-TR axis I disorders (SCID^49^) was additionally used to confirm SZ diagnosis and the absence of any Axis I diagnoses in TD. Means and standard deviations, as well as group comparison tests of the above-mentioned instruments, are given in Table 1.

### Imaging data acquisition, preprocessing, and motion correction

All resting-state fMRI scans lasted 7.5 min and were collected using a Siemens Skyra 3 T scanner at the ONRC. Participants lay still, with eyes open, while fixating on a centrally presented cross. Blood oxygenation level-dependent (BOLD) signal was obtained with a T2*-weighted echo-planar (EPI) sequence: RT = 475 msec, TE = 30 msec, flip-angle = 60°, 48 slices, multiband (8), interleaved slice order, 3 mm^3^ voxels.

Pre-processing of structural MRI data was done using fMRIPrep v20.2.6^50^, which is based on Nipype 1.7.0^51,52^. The pipeline included the following steps: correction for intensity non-uniformities using field maps^53^, skull-stripping using ANTs 2.3.3^54^ with OASIS30ANTs as target template, segmentation using the FAST algorithm from FSL 5.0.9^55^, and normalization using antsRegistration (ANTs 2.3.3). Functional scans were co-registered with FLIRT (FSL 5.0.9^56^,) using nine degrees of freedom and spatiotemporal filtering was performed using MCFLIRT (FSL 5.0.9^57^). Finally, a slice-time correction was applied^58^.

Motion artifacts were first removed using non-aggressive ICA-AROMA^59^, following smoothing with a 6 mm FWHM kernel. Detrending was then performed using DiCER^60^. Finally, frame-wise displacement (FD) motion parameters were computed according to the FSL library algorithm^61^ and later used as covariates to check that our results were not biased by potential motion artifacts.

### Data analysis

The INT analysis steps described in ref. ^24^ and implemented in ref. ^25^ were followed. First, an autocorrelation function (ACF) was calculated for each voxel. The ACF measures how data points in a time series are related to each other, or in other words, the self-similarity of the rsfMRI BOLD signal. First, we set a maximum time lag of 20 s and divide it into smaller, incremental timesteps/time lags for each second. At each timestep, we correlate the preceding and the current signal and proceed thus until the value of the correlation turns negative. Finally, the resulting positive autocorrelation values at each voxel were summed up, and this value was then multiplied by the TR, thus resulting in the final INT index.

Whole-brain analyses were performed using the SPM12 software (http://www.fil.ion.ucl.ac.uk/spm/) while the regions of interest (ROIs) were defined using the MARSBAR toolbox. Further statistical analyses were performed in R 5.263 software.

The ROIs were defined as 6 mm radius spheres centered around the peak MNI coordinates reported by Watanabe et al.^24^ and Uscătescu et al.^25^. The peak coordinates of all 13 ROIs are shown in Table 4 (note that regions derived from Watanabe et al.^24^ are prefixed with ‘W’ and those from Uscătescu et al.^25^ with ‘U’).

**Table 4:** Peak MNI coordinates reported by refs. ^24^ and^25^, around which the ROIs of the current study were defined.

MNI Coordinates and Names of ROIs				
ROI		MNI Coordinates		
ROI Acronym	ROI Description	X	Y	Z
Watanabe et al.^24^				
W_rPCG	right Post-Central Gyrus	58	–14	44
W_lPCG	left Post-Central Gyrus	–58	–14	40
W_rMTG	right Middle Temporal Gyrus	60	2	26
W_lMTG	left Middle Temporal Gyrus	–70	–26	6
W_rIOG	right Inferior Occipital Gyrus	52	–74	6
W_rIPL	right Inferior Parietal Lobule	50	–44	32
W_rMidIns	right Middle Insula	50	10	4
W_rCaud	right Caudate	14	20	12
Uscătescu et al.^25^				
U_rOccFusG	right Occipital Fusiform Gyrus	27	–88	14
U_lSupOccG	left Superior Occipital Gyrus	–15	–91	19
U_rSupOccG	right Superior Occipital Gyrus	18	–79	25
U_lLatOccC	left Lateral Occipital Cortex	–42	–70	1
U_rPostCenG	right Post-Central Gyrus	42	–31	64

## Supplementary information

Table
