# Supplementary material for: Intrinsic neural timescales in autism spectrum disorder and schizophrenia. A replication and direct comparison study
Source: Schizophrenia (Heidelb). 2023 Mar 30;9(1):18. doi: 10.1038/s41537-023-00344-1 (PMC10063601; doi:10.1038/s41537-023-00344-1)
Supplement: Supplementary file 1 — Table [file 41537_2023_344_MOESM1_ESM.docx]

Supplement

**Table 1.** Peak MNI coordinates, cluster size, uncorrected *p* values and F or T values for the exploratory results depicted in Figure 2 B and C.

**Peak Voxel Coordinates of Exploratory Analyses**

|  | MNI | | |  |  |  |
| --- | --- | --- | --- | --- | --- | --- |
| TD v. ASD v. SZ | X | Y | Z | Number of voxels | *p* uncorr (cluster level) | F value |
| right inferior temporal gyrus | 42 | 0 | -42 | 2 | .74 | 10.13 |
| right precentral gyrus | 46 | 0 | 48 | 21 | .24 | 9.68 |
| right fusiform gyrus | 44 | -52 | -20 | 5 | .58 | 8.76 |
| left inferior occipital gyrus | -42 | -72 | 8 | 13 | .35 | 8.59 |
| left supramarginal gyrus | -42 | -32 | 42 | 11 | .4 | 7.98 |
| right fusiform gyrus | 30 | -4 | -38 | 1 | .83 | 7.31 |
| ASD > TD |  |  |  |  |  | T value |
| right inferior temporal gyrus | 42 | -2 | -42 | 12 | .43 | 3.86 |
| right entorhinal area | 20 | 0 | -38 | 4 | .67 | 3.46 |
| right temporal pole | 48 | 6 | -46 | 2 | .78 | 3.44 |
| left fusiform gyrus | -28 | -6 | -46 | 5 | .63 | 3.34 |
| right inferior temporal gyrus | 50 | -6 | -38 | 1 | .85 | 3.24 |
| right cerebellum | 44 | -46 | -42 | 1 | .85 | 3.24 |
| left middle temporal gyrus | -66 | -60 | 8 | 1 | .85 | 3.18 |
| ASD > SZ |  |  |  |  |  |  |
| left middle frontal gyrus | -40 | 36 | 24 | 16 | .43 | 4.06 |
| left superior parietal lobule | -32 | -62 | 68 | 5 | .68 | 3.83 |
| right superior parietal lobule | 20 | -64 | 70 | 4 | .71 | 3.76 |
| right inferior temporal gyrus | 42 | 0 | -42 | 1 | .86 | 3.74 |
| right superior parietal lobule | 14 | -62 | 68 | 7 | .61 | 3.66 |
| right precentral gyrus | 44 | 0 | 54 | 20 | .37 | 3.65 |
| left precentral gyrus | -48 | -10 | 60 | 5 | .68 | 3.59 |
| right inferior frontal gyrus (opercular part) | 58 | 20 | 2 | 3 | .76 | 3.53 |
| left precuneus | 0 | -72 | 56 | 7 | .61 | 3.52 |
| right superior parietal lobule | 10 | -56 | 64 | 3 | .76 | 3.48 |
| right middle occipital gyrus | 34 | -86 | 20 | 7 | .61 | 3.47 |
| right inferior temporal gyrus | 30 | 0 | -42 | 1 | .88 | 3.45 |
| left temporal pole | -40 | 22 | -42 | 1 | .88 | 3.45 |
| left superior parietal lobule | -26 | -60 | 72 | 7 | .61 | 3.44 |
| right precuneus | 2 | -46 | 72 | 2 | .81 | 3.41 |
| left inferior occipital gyrus | -52 | -74 | -8 | 1 | .88 | 3.36 |
| right fusiform gyrus | 32 | -2 | -38 | 2 | .81 | 3.35 |
| left fusiform gyrus | -44 | -50 | -16 | 3 | .76 | 3.33 |
| right superior parietal lobule | 42 | -44 | 66 | 1 | .88 | 3.31 |
| right supramarginal gyrus | 52 | -24 | 42 | 3 | .76 | 3.28 |
| left superior frontal gyrus | -8 | 68 | 24 | 1 | .88 | 3.27 |
| left inferior occipital gyrus | -42 | -74 | 8 | 1 | .88 | 3.23 |
| right superior parietal sulcus | 10 | -56 | 74 | 1 | .88 | 3.22 |
| TD > SZ |  |  |  |  |  |  |
| right precentral gyrus | 46 | 0 | 48 | 60 | .07 | 4.37 |
| right fusiform gyrus | 44 | -52 | -20 | 35 | .16 | 4.13 |
| left inferior occipital gyrus | -42 | -72 | 8 | 129 | .01 | 4.12 |
| left inferior occipital gyrus | -54 | -74 | 0 |  |  | 3.64 |
| left supramarginal gyrus | -42 | -32 | 42 | 60 | .07 | 3.82 |
| left superior occipital gyrus | -16 | -90 | 36 | 19 | .29 | 3.68 |
| right precentral gyrus | 60 | 12 | 28 | 16 | .33 | 3.67 |
| right postcentral gyrus | 68 | -10 | 16 | 4 | .64 | 3.64 |
| left superior frontal gyrus | -14 | 52 | 44 | 7 | .53 | 3.57 |
| right superior frontal gyrus | 28 | 38 | 50 | 2 | .76 | 3.51 |
| right middle frontal gyrus | 38 | 10 | 28 | 11 | .42 | 3.49 |
| right precentral gyrus | 2 | -28 | 66 | 10 | .45 | 3.46 |
| right superior parietal lobule | 10 | -54 | 72 | 10 | .45 | 3.45 |
| right middle frontal gyrus | 34 | 4 | 66 | 2 | .76 | 3.42 |
| left precentral gyrus | -12 | -22 | 80 | 5 | .6 | 3.40 |
| left superior parietal lobule | -30 | -62 | 62 | 7 | .53 | 3.38 |
| right superior frontal gyrus | 22 | -2 | 76 | 3 | .7 | 3.35 |
| right superior frontal gyrus | 8 | 16 | 68 | 1 | .84 | 3.31 |
| left central operculum | -40 | -18 | 20 | 2 | .76 | 3.29 |
| left superior frontal gyrus | -12 | -10 | 78 | 3 | .7 | 3.28 |
| right precentral gyrus | 6 | -32 | 76 | 1 | .84 | 3.23 |
| left superior frontal gyrus | -2 | 58 | 8 | 1 | .84 | 3.22 |
| left superior frontal gyrus | -20 | 50 | 44 | 1 | .84 | 3.21 |
| right precentral gyrus | 52 | 10 | 30 | 1 | .84 | 3.21 |
| right middle frontal gyrus | 38 | 4 | 64 | 1 | .84 | 3.21 |
| right superior parietal lobule | 46 | -42 | 64 | 1 | .84 | 3.20 |
| left inferior occipital gyrus | -48 | -78 | -4 | 2 | .76 | 3.20 |
| right occipital fusiform gyrus | 42 | -64 | -16 | 1 | .84 | 3.20 |
| left inferior occipital gyrus | -40 | -68 | -4 | 1 | .84 | 3.19 |
| right middle frontal gyrus | 48 | 32 | 32 | 1 | .84 | 3.19 |
| right inferior occipital gyrus | 54 | -66 | 6 | 1 | .84 | 3.18 |
| right superior frontal gyrus | 12 | 38 | 26 | 1 | .84 | 3.18 |
